# MGMTai: O6-methylguanine-DNA methyltransferase (*MGMT*) methylation prediction in isocitrate dehydrogenase (*IDH*)-wild type glioblastoma to direct temozolomide therapy

**DOI:** 10.1093/noajnl/vdag103

**Published:** 2026-04-16

**Authors:** Patricia Pittman, Chen Sun, Timothy Samec, Christian Davidson, David Bryant, Iyad Alnahhas, Michael Glantz, Robert Hahn-Lowry, David Spetzler, Daniel Magee, Theodore Nicolaides

**Affiliations:** Caris Life Sciences, Phoenix, Arizona, USA; Caris Life Sciences, Phoenix, Arizona, USA; Caris Life Sciences, Phoenix, Arizona, USA; Caris Life Sciences, Phoenix, Arizona, USA; Caris Life Sciences, Phoenix, Arizona, USA; Division of Neuro-Oncology, Department of Neurology, Thomas Jefferson University, Philadelphia, Pennsylvania, USA; Department of Neurosurgery, Penn State Milton S. Hershey Medical Center, Hershey, Pennsylvania, USA; Department of Oncology, Penn State Milton S. Hershey Medical Center, Hershey, Pennsylvania, USA; Caris Life Sciences, Phoenix, Arizona, USA; Caris Life Sciences, Phoenix, Arizona, USA; Caris Life Sciences, Phoenix, Arizona, USA; Caris Life Sciences, Phoenix, Arizona, USA

**Keywords:** glioblastoma, MGMT methylation, molecular classification, predictive biomarker, temozolomide

## Abstract

**Background:**

*MGMT* promoter methylation status has been utilized as a predictor of response to temozolomide in patients with *IDH*-wildtype glioblastoma (GBM). Traditional methods of methylation status identification include methylation-specific polymerase chain reaction and pyrosequencing (PyroSeq). Though widely used, each method has disadvantages with respect to determining methylation cut-off values, tumor content required for evaluation, prognostic accuracy, and financial expense.

**Methods:**

We have developed a method of *MGMT* classification using artificial intelligence and a large clinicogenomic database of 5841 GBM patients. We evaluate the performance of this novel classification strategy for predicting temozolomide treatment efficacy in comparison to PyroSeq techniques.

**Results:**

MGMTai reliably predicted *MGMT* methylation status in GBM patients with available PyroSeq data. Comparative bucketing of methylation status based on MGMTai and PyroSeq yielded high sensitivity and positive predictive value concordance, though new *MGMT* promoter methylation (*MGMT^met^*) status percentages were drawn according to PyroSeq methylation percent values. Overall survival with temozolomide (TMZ) treatment was comparable between PyroSeq and MGMTai; however, MGMTai by decile and MGMTai stratified into 3 scoring buckets yielded more distinct and predictive survival patterns with increasing MGMTai score compared to PyroSeq.

**Conclusion:**

Implementation of an AI-based molecular classification system, MGMTai, can better describe *MGMT* methylation status in comparison to the traditional PyroSeq method. Patient *MGMT^met^* status by MGMTai scoring drew more distinct survival curves and predicted TMZ-treated GBM patient survival more efficiently, less expensively, and with better precision and reproducibility than PyroSeq.

Key Points

- MGMTai utilizes already available gene sequencing data at Caris Life Sciences to reliably predict *MGMT* methylation status using an AI-based method.
- MGMTai score buckets captured patient survival trends dependent on *MGMT^met^* characterization with better population discrimination compared to PyroSeq.
- In GBM patients treated with TMZ, overall survival was more accurately predicted using MGMTai than PyroSeq.

Importance of the Study

AI-driven molecular characterization of *MGMT* methylation draws more reliable methylation status cutoff values and more discriminative overall survival curves while reducing assay resources compared to traditional pyrosequencing.

As the most frequent malignant brain tumor in adults, isocitrate dehydrogenase (*IDH)1/2*-wildtype (WT) Glioblastoma (GBM) presents major clinical challenges, with the median survival time in the range of 12 months irrespective of therapeutic interventions.^1^ Standard management includes maximum safe tumor resection, radiotherapy with concurrent alkylating chemotherapy (temozolomide; TMZ), and adjuvant, postradiation alkylating chemotherapy. Previous studies examined the efficacy of platinum-based therapies, carboplatin, and cisplatin, but found enhanced toxicity without improved clinical benefit.^2^ Thus, the standard chemotherapeutic intervention remains TMZ. Unfortunately, nearly 50% of patients do not respond to TMZ and, in TMZ responders, nearly all develop TMZ resistance and disease recurrence.^3^ As such, there is a desperate need to identify patients who would preferentially respond to TMZ, allowing likely nonresponders to participate in clinical trials designed to identify more effective treatment alternatives.

Promoter methylation of O6-methylguanine-DNA methyltransferase (*MGMT*) is a well-established predictive biomarker for GBM patient response to TMZ. The use of *MGMT* promoter methylation (*MGMT^met^*) as a predictive biomarker has been commonplace in neuro-oncology for over 15 years,^4^ with substantial evidence of improved TMZ treatment efficacy for GBM patients over patients with low *MGMT^met^*.^5–7^ Traditional methods of *MGMT^met^* testing have included nonquantitative methylation-specific polymerase chain reaction (MSP),^4^^,^^8^ quantitative MSP (qMSP),^8^^,^^9^ pyrosequencing (PyroSeq),^10^ quantitative real-time polymerase chain reaction (qRT-PCR),^11^ and immunohistochemistry (IHC).^12^ These multiple available testing strategies for *MGMT^met^* status have resulted in marked variation of *MGMT^met^* status between methods. Specifically, IHC and MSP were shown to be highly discordant for *MGMT^met^* in brain tumors across a meta-analysis of 52 studies.^13^ Evaluation of IHC and PyroSeq was also compared by Wang et al,^14^ revealing a concordance rate of only 30.8%. This inability to produce consistent and reliable outcomes results in increased cost, treatment delays, inaccurate discussions of prognosis, and potential errors in therapeutic decision-making.

In this work, we describe a novel predictive model, MGMTai, generated using AI-based exome and transcriptome analysis from 5841 patients meeting eligibility criteria, in concert with a clinical database. We show the ability of MGMTai to reproduce, support, and provide additional clinically relevant *MGMT^met^* detail beyond the standard “methylated/not methylated” dichotomy in current testing methods, specifically PyroSeq. We describe the clinical utility of this system compared to currently accepted methods of *MGMT^met^* classification. We also present encouraging positive predictive value (PPV) and sensitivity data that demonstrate the robustness of MGMTai. Further, using a classification system based on *MGMT^met^* scores, the predictive performance and clinical utility of this model are demonstrated through analysis of overall survival (OS) in GBM patients treated with TMZ and a prospective GBM analysis.

## Methods

### Tissue Acquisition

Molecular profiling was performed on over 484 000 de-identified tumors at Caris Life Sciences (Phoenix, AZ, United States), a College of American Pathologists (CAP)/Clinical Laboratory Improvement Amendments (CLIA)-certified laboratory. Of those, 11 579 were GBM patient samples eligible for sequencing, training, and testing of the MGMTai model. Hematoxylin and eosin-stained formalin-fixed, paraffin-embedded (FFPE) slides of the patient’s tumor underwent review by a board-certified pathologist or trained pathologist assistant. Tumor enrichment was achieved by harvesting targeted tissue using manual microdissection techniques. A minimum of 20% tumor nuclei content within the microdissection was required to perform subsequent sequencing experiments, and a minimum of 50% tumor content was required for *MGMT* PyroSeq.

### Next-Generation Sequencing/Whole Transcriptome Sequencing

RNA from over 484 000 patient samples, of which 7,473 of the eligible 11 579 GBM cases, was extracted using an RNA FFPE Extraction Kit (Qiagen, Hilden, Germany), and RNA quality and quantity were determined using the Agilent TapeStation (Agilent Technologies, Santa Clara, CA, United States). A SureSelect Human All Exon v7 panel of biotinylated RNA baits (Agilent Technologies) was hybridized to the synthesized and purified cDNA targets, and the bait-target complexes were amplified in a postcapture PCR reaction. The resultant libraries were quantified and normalized, and the pooled libraries were denatured and diluted. Library preparation, quantification, normalization, and pooling were performed using the Bravo Automated Liquid Handling Platform and the Agilent Tape Station 4200 (Agilent Technologies). Whole transcriptome sequencing (WTS) was performed on the Novaseq 6000 System (Illumina, San Diego, CA, United States) (RRID: SCR_016387).

### MI Tumor Seek Hybrid Assay

Total nucleic acid (TNA) from 4,106 of 11 579 eligible GBM tumor samples was auto-extracted using a MagMax FFPE DNA/RNA Ultra extraction kit (ThermoFisher Scientific, Waltham, MA, United States). To perform simultaneous DNA and RNA sequencing using the Caris Life Sciences Molecular Intelligence (MI) Tumor Seek Hybrid assay, library preparation was performed on the Bravo Automated Liquid Handling Platform (Agilent, Santa Clara, CA, United States) using KAPA Library Prep reagents (Roche, Indianapolis, IN) and custom cDNA primers (IDT, Coralville, IA, United States; GeneLink, Elmsford, NY, United States). Custom KAPA bait panels were designed to enrich for 720 clinically relevant genes at high coverage and high read depth, and an additional >20 000 genes at lower depth, along with SNP and pathogen panels (Roche). Sequencing was performed on the NovaSeq 6000 System (Illumina) using recommended reagents. The average sequencing depth of this assay is 230× for 20 859 genes (whole exome), 1000× for 720 genes with known and potential clinical relevance, and 1500× for 230 reportable genes. RNA is sequenced to a minimum of 1.37 million total mapped reads. Sequencing data were extracted into split FASTQ files (RNA and DNA) for further processing using Caris’s bioinformatics pipeline. For RNA, Spliced Transcripts Alignment to a Reference (STAR) software was used for alignment, trimming, and fusion detection using the RNA FASTQ files from the TNA split pipeline.^15^ Transcripts per million (TPM) molecules were generated using the Salmon expression pipeline.^16^ RNA data normalization was performed to collate WTS and MI Tumor Seek Hybrid expression values (Figure S1).

### Pyrosequencing

*MGMT* PyroSeq was performed to assess the methylation status of the *MGMT* promoter CpG sites (CpGs 74-78)^17^^,^^18^ to predict the clinical response of GBM to TMZ. DNA was extracted from FFPE tissue (≥50% tumor nuclei required) and treated with bisulfite to convert cytosine, but not 5-methylcytosine, to uracil. A DNA protect buffer was added to minimize DNA degradation during bisulfite conversion. After bisulfite treatment, the DNA was purified using a column designed for minimal elution volumes. The bisulfite-converted DNA was then amplified using biotin-labeled primers for 5 different methylation sites in exon 1 of *MGMT*. PCR products were bound to beads via streptavidin, denatured, washed, and annealed to sequencing primers. PyroSeq was performed using the PyroMark system (Qiagen, Hilden, Germany). Samples with ≥9% methylation were considered hypermethylated, ≥7% and <9% was considered equivocal, and <7% was considered unmethylated for model training. Pathologists were masked to all clinical characteristics, including OS.

### CODEai Clinical Data Portal

Real-world OS information was obtained from insurance claims data. Overall survival was calculated from the start of treatment with (therapy) until last contact. Patients showing an initiation of TMZ prior to the biopsy date were excluded from survival analyses in order to limit the analysis to treatment-naïve tumors. Patients who began TMZ >100 days after the biopsy date or who received fewer than 3 unique administrations of TMZ were also excluded from survival analyses. The endpoint for OS was either the last contact date or known death date. Patients with a last contact date within 100 days of the most recent clinical claim date in the full real-world data collection were considered censored, and others were presumed deceased. Internal Caris analysis of a subset of patients with recorded deaths in the insurance claims database showed that OS differed by <1 month compared to the inferred method using last contact.

### Cohort Selection Criteria

Patient cohorts were filtered from the over 484 000 samples available in the Caris Life Sciences data repository to 5841 that represented the final study cohort. Filtration criteria followed a progression of identifying samples of *IDH1/2*^WT^ GBM origin where patients were at least 50 years of age and removal of samples that did not have comprehensive WES and WTS data with a confirmatory *MGMT* methylation results via PyroSeq (*n* = 11 579). Of these, 10 021 had at least 1 TMZ treatment, and only 5841 had sufficient clinical claims data available to capture TMZ administration and a minimum of 9-month follow-up. Model training was performed on this final cohort of 5841 patient samples. The OS prediction cohort was further filtered by patients with at least 100 days of TMZ therapy and at least 3 TMZ cycles (*n* = 2252). The prospective validation cohort of 3464 patients was selected solely on the availability of PyroSeq data over a 9-month period beginning February 2025 and is not included in OS analyses.

### Classifier and Other Algorithmic Methods

The MGMTai classifier was built using Light Gradient Boosted Machine learning (LightGBM) model, employing a nested 5-fold cross-validation strategy. The dataset was partitioned into 5 folds, with each outer fold using approximately 80% of samples for model development and 20% held out as an independent test set for performance estimation (ie prediction was generated only on the outer held-out fold). Within each outer training split, an inner 5-fold cross-validation procedure was performed for hyperparameter tuning and regularization, including selection of early stopping parameters in LightGBM. The raw next-generation sequencing (NGS)-derived feature space comprised >20 000 gene expression measures and >20 000 copy-number features, which were subsequently filtered and curated to the final candidate set used for model development. The candidate feature set comprised 1110 variables: 711 gene expression, 184 mutation, and 215 copy-number features, selected for relevance to central nervous system cancers.

To reduce model complexity and mitigate overfitting, we first trained a LightGBM model using all available features and then selected the top 100 features based on LightGBM feature-importance rankings. Model performance was comparable between the all-feature model (PPV/negative predictive value [NPV] = 0.838/0.838) and the top-100-feature model (PPV/NPV = 0.835/0.846), supporting the use of the reduced feature set for subsequent cross-validation and prospective deployment. The final model utilized the top 100 DNA and RNA features derived from sequencing of GBM tumor data (Table S1), including 6 gene copy number variations and 94 gene expressions (TPM). Clinical outcomes data were not involved in model training or evaluation. During model training, validation, and cross-validated testing, only samples classified as methylated or unmethylated by MGMT PyroSeq (using predefined thresholds reported clinically) were included; samples deemed equivocal by PyroSeq were excluded from these steps. Following an accuracy assessment on the cross-validated data, a model was trained using all available nonequivocal samples and was deployed to predict methylation status on prospective cases, including PyroSeq-equivocal cases, which were evaluated only after model finalization.

### Statistical Analysis

Overall survival estimates were obtained using the Kaplan-Meier method, and hazard ratios for survival were calculated using the multivariate Cox Proportional Hazards model. *P* values were calculated using the log-rank test, where *p* ≤ .05 was defined as statistically significant. Positive predictive value and sensitivity tables were derived from 2 × 2 contingency tables, using MGMTai as the index test and PyroSeq as the clinical standard.

### Ethics

This study was conducted in accordance with the guidelines of the Declaration of Helsinki, Belmont Report, and US Common rule. In keeping with 45 CFR 46.104(d)(4), this study was performed utilizing retrospective, deidentified clinical data and is considered IRB exempt with waivers of patient informed consent. Waiver of patient consent and exempt status were determined by wcg IRB.

## Results

### GBM Patient Demographics

Though 11 579 GBM cases were sequenced and available for training and testing of the MGMTai model, only 5841 made up the final cohort with clinical claims data available, including a record of prior TMZ treatment and at least 9 months of follow-up data. Figure S1 displays expression normalization of the full 11 579 cases, including 35% of which were analyzed via MI Tumor Seek Hybrid, and an initial analysis of *MGMT* RNA expression compared against the standard PyroSeq methylation status result. The 5841 cases, described in Table 1, were predominantly male (58.2%), White race (86.5%), and of non-Hispanic/Latino ethnicity (92.9%). Discrepancies in the total number of cases from 5841 to the 4355 reporting race and 4696 reporting ethnicities are accounted for by optional patient responses to these demographic variables. Glioblastoma-specific molecular markers of any kind were found in 87.3% of patients, with individual markers of *TERT* mutation found in 80.4%, *EGFR* amplification in 35.0%, and chromosome 7 gain/10 loss (+7/−10) in 36.1% of patients. Eligibility criteria required these 5841 cases to have *MGMT*^met^ status as determined by PyroSeq prior to further analysis. Of this final cohort, 42.7% were considered hypermethylated, 55.0% not methylated, and 2.3% equivocal (Table 2A). Corresponding median OS (mOS) for each group according to clinical claims data were 16.3, 11.9, and 13.1 m, respectively (Table 2A).

**Table 1.** Cohort demographics for patients with *IDH^WT^* GBM with clinical claims data including TMZ treatment

*IDH^WT^* GBM—TMZ Tx (*N* = 5841)		
Characteristic		Percentage (*n*)
Median age [25, 75 percentiles]		66 [60, 72]
Sex	Male	58.2% (3398/5841)
	Female	41.8% (2443/5841)
Race	White	86.5% (3769/4355)
	Black/AA	6.6% (288/4355)
	Asian/PI	2.5% (111/4355)
	Other	4.3% (187/4355)
Ethnicity	Not Hispanic or Latino	92.9% (4399/4696)
	Hispanic or Latino	7.1% (332/4696)
GBM markers	*TERT* mutation	80.4% (4697/5841)
	*EGFR* amplified	35.0% (2047/5841)
	Chr +7/−10	36.1% (2108/5841)
	Any	87.3% (5100/5841)

Abbreviations: GBM, glioblastoma; TMZ, temozolomide.

**Table 2.** Cohort *MGMT* methylation status via pyrosequencing and MGMTai with corresponding PyroSeq median OS for patients with *IDH^WT^* GBM with clinical claims data including TMZ treatment (A) and concordance between MGMTai and PyroSeq (B)

A				
IDH^WT^ GBM—TMZ Tx (*N* = 5841)				
PyroSeq classification	Percentage (*n*)	mOS (months)	MGMTai bucket	Percentage (*n*)
Hypermethylated	42.7% (2494)	16.3	(0.8-1]	27.5% (1606)
Equivocal	2.3% (137)	13.1	(0.2-0.8]	29.9% (1752)
Not methylated	55.0% (3210)	11.9	(0.0-0.2]	42.5% (2483)

B				
Concordance of MGMTai vs PyroSeq		*MGMT^met^* status via PyroSeq		
		Not methylated (sensitivity; PPV)	Equivocal (sensitivity; PPV)	Hypermethylated (sensitivity; PPV)
MGMTai score	(0-0.2]	0.705; 0.911	0.175; 0.01	0.079; 0.08
	(0.2-0.8]	0.249; 0.457	0.467; 0.037	0.356; 0.507
	(0.8-1]	0.046; 0.092	0.358; 0.031	0.565; 0.878

Abbreviations: GBM, glioblastoma; MGMT, O6-methylguanine-DNA methyltransferase; *MGMT^met^*, *MGMT* promoter methylation; mOS, median overall survival; OS, overall survival; PPV, positive predictive value; PyroSeq, pyrosequencing; TMZ, temozolomide.

### MGMTai Exhibits High Concordance with PyroSeq

**Figure 1.**
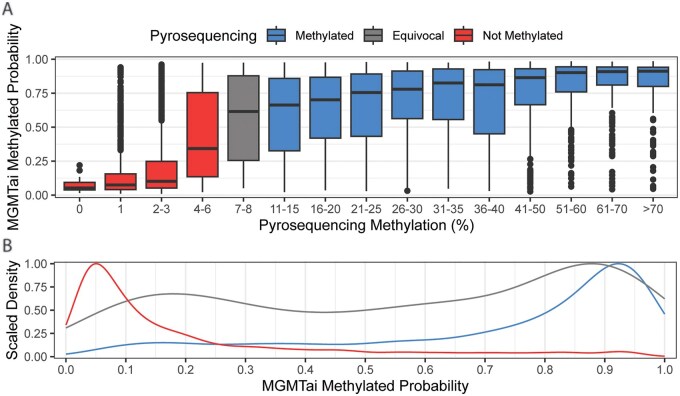
MGMTai prediction probability in GBM patients with pyrosequencing data. MGMTai probability of assigning not methylated (red), equivocal (gray), or hypermethylated (blue) *MGMT^met^* status according to pyrosequencing (PyroSeq) methylation percentage (A). Scaled density of GBM patients assigned to *MGMT^met^* status as not methylated (red), equivocal (gray), or hypermethylated (blue) (B). GBM, glioblastoma; MGMT, O^6^-methylguanine-DNA methyltransferase; *MGMT^met^*, *MGMT* promoter methylation.

Glioblastoma patients with PyroSeq data were classified as either hypermethylated, equivocal, or not methylated by methylation percentage score (Figure 1A). These PyroSeq data relative to *MGMT* expression are presented in Figure S2. Relative to these PyroSeq classifications, the probability of MGMTai methylation classification was given by scaled densities, showing specificity to tail ends where heavy densities of cases are expected to be assigned (Figure 1B). This pattern is similar to that seen in PyroSeq classification of methylation status, with the bulk of cases either methylated or not methylated, with a central distribution of equivocal status shown in Figure 1A and B. Cutoffs for each classification were 0%-6% (not methylated), >7%-9% (equivocal), and >9% (hypermethylated). Using the same cohort of patients, the MGMTai classifier, using 100 features according to importance rankings of DNA and RNA (Table S1), reported scores from 0.00 to 1.00, with increasing values characterizing higher probability of methylation. Bucketing of MGMTai scores was performed using a 3-way bucket, where ≤0.2 represented predicted nonmethylated *MGMT*, (0.2-0.8] represented predicted equivocal *MGMT*, and >0.8 represented predicted hypermethylated *MGMT*, selected as uniform cutoffs to split the population into 3 roughly equal-sized categories and enabled concordance against *MGMT^met^*. Of the 5841 patient samples analyzed, 42.5% (2483) fell into the [0-0.2) score bucket, 29.9% (1752) in the (0.2-0.8] score bucket, and 27.5% (1606) in the (0.8-1] score bucket (Table 2A), where [0-0.2) displayed the most similar distribution to the corresponding PyroSeq characterization (Not Methylated, 55.0% vs [0-0.2), 42.5%). Sensitivity analysis exhibited values of 0.565 for the (0.8-1] MGMTai bucket to PyroSeq hypermethylation status and 0.705 for (0-0.2] bucket to PyroSeq Not Methylated Status (Table 2B). Positive predictive value (Table 2B) reported similar results, with PPV values of 0.878 for (0.8-1] and PyroSeq hypermethylated and 0.911 for (0-0.2] and PyroSeq Not Methylated.

### Clinical Outcomes in TMZ-Treated GBM Patients in PyroSeq and MGMTai by MGMT^met^ Bucketing

**Figure 2.**
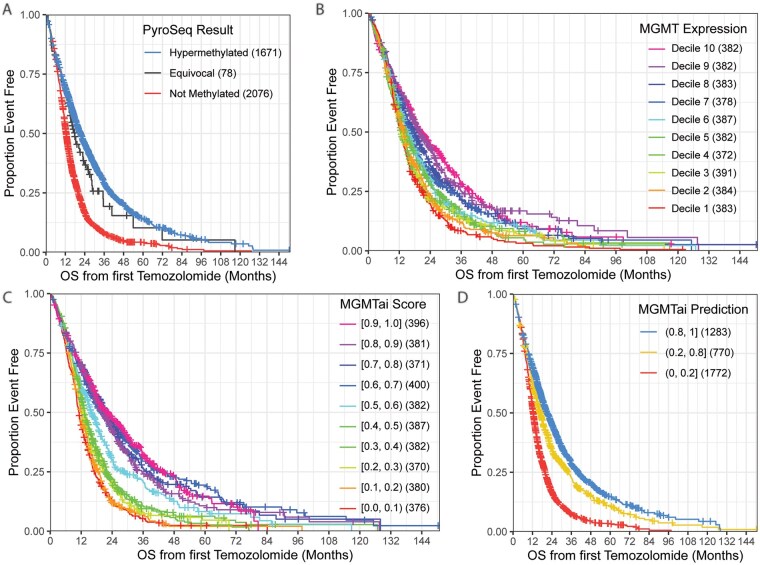
Overall survival (OS) from first temozolomide (TMZ) treatment by traditional pyrosequencing, *MGMT* expression, and MGMTai. OS in months post-TMZ by *MGMT^met^* status determined by pyrosequencing (PyroSeq) as hypermethylated, equivocal, or not methylated (A), MGMT expression by decile, where expression decreases with increasing decile (B), MGMTai score by decile (C), and MGMTai by final bucketing (D). MGMT, O^6^-methylguanine-DNA methyltransferase; *MGMT^met^*, *MGMT* promoter methylation.

Traditional PyroSeq results are reported as “hypermethylated” with *MGMT^met^* of >8%, “equivocal” with *MGMT^met^* of >6%-8%, or “not methylated” with *MGMT^met^* of 0%-6%. Shown in Figure 2A, this categorization assigns 43.7% of patient tissue as hypermethylated (1671/3825) and exhibited improved OS from the first TMZ. *MGMT* by expression showed lower *MGMT* expression values (deciles 8-10) were associated with longer OS from first TMZ administration (Figure 2B). MGMTai classification by decile (Figure 2C) visually exhibited clearer bucket separations, with score values below 0.5, score values between 0.5 and 0.6, and score values above 0.6 having the clearest delineations before bucket consolidation. Altogether, higher MGMTai scores, above 0.8, were associated with longer OS from first TMZ. From these data, MGMTai buckets were collapsed as shown in Figure 2D, at score values of [0-0.2), [0.2-0.8), and [0.8-1), the same buckets used to quantify sensitivity, specificity, PPV, and NPV against the gold standard. Case distribution by MGMTai score with bucketing delineation and OS by PyroSeq *MGMT^met^* percent are shown in Figure S3A and B.

### MGMTai Predicts OS from First TMZ Consistent with PyroSeq Analyses

**Figure 3.**
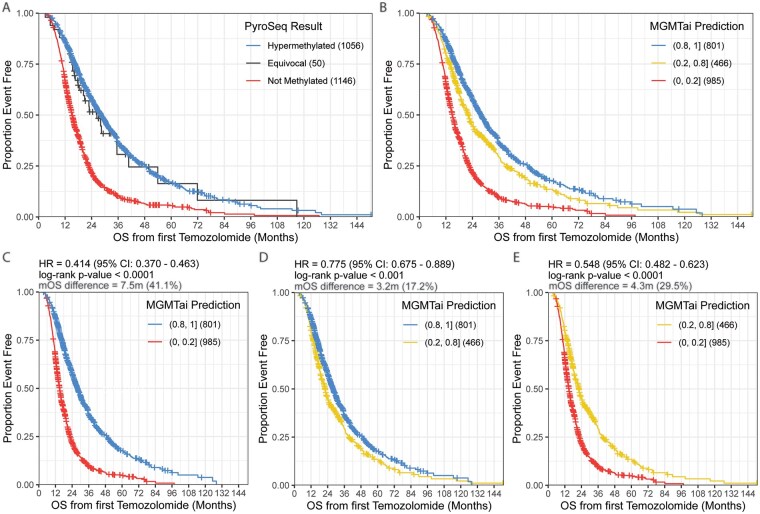
Overall survival (OS) from first temozolomide (TMZ) treatment by traditional pyrosequencing or MGMTai. OS in months post-TMZ by *MGMT^met^* status determined by pyrosequencing as hypermethylated, equivocal, or not methylated (A) or MGMTai as methylation score buckets (B). OS comparison between MGMTai predicted methylation score buckets (0.8, 1] and (0, 0.2] (C), (0.8, 1] and (0.2, 8] (D), (0, 0.8] and (0, 0.2] (E). Statistical significance where *p* ≤ .05. MGMT, O^6^-methylguanine-DNA methyltransferase; *MGMT^met^*, *MGMT* promoter methylation.

*MGMT^met^* via PyroSeq and MGMTai OS curves were cal­culated for the final patient cohort as determined by neuropathologist review to include patients over 50 years of age with only high-grade, *IDH1/2*^WT^GBM with clinical outcomes data following at least 100 days of TMZ therapy and at least 3 TMZ cycles. A total of 2252 patients meeting those criteria were characterized via PyroSeq (Figure 3A), exhibiting similar results as previously described, with hypermethylation exhibiting enhanced OS (mOS = 22.3 months) over equivocal (mOS = 18.8 months) and not methylated (mOS = 14.7 months) *MGMT* status. The MGMTai classifier also yielded a near-identical pattern, with a score of (0.8-1] representing the best surviving patient cohort (mOS = 22.0 months) having prior TMZ therapy (Figure 3B) over scores of (0.2-0.8] (mOS = 18.8 months) and (0.0-0.2] (mOS = 14.5 months). Cross-comparison of each score bucket reported an improved mOS percentage difference of 41.1% between MGMTai scores of (0.8-1] and (0.0-0.2] (7.5 months, Figure 3C), an improved mOS percentage of 17.2% between (0.8-1] and (0.2-0.8] (3.2 months, Figure 3D), and an improved mOS percentage of 29.5% between (0.2-0.8] and (0.0-0.2] (4.3 months, Figure 3E), where all differences were statistically significant (*p* ≤ .0001). Overall survival for molecularly defined GBM, as determined by presence of *TERT* mutation, *EGFR* amplification, and Chr +7/−10 modification, is presented in Figure S4.

### Prospective Validation

**Figure 4.**
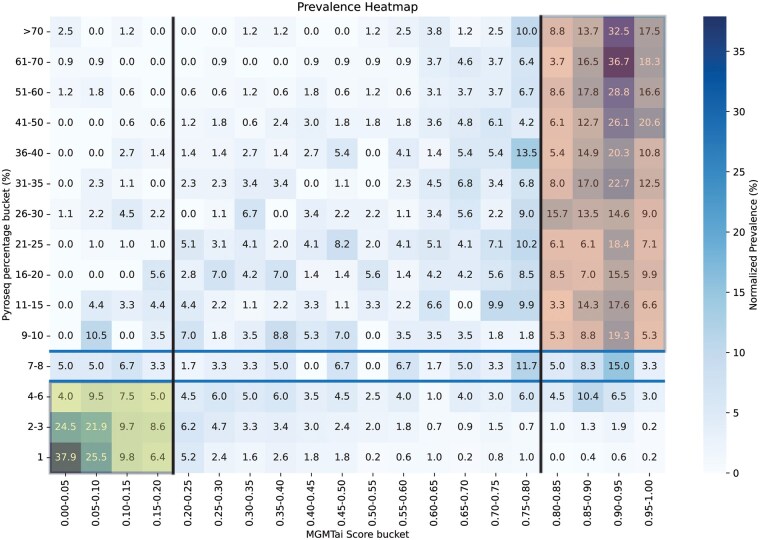
Prospective validation of MGMTai by case prevalence relative to PyroSeq. MGMTai prospectively predicted methylation score of 2,772 *IDH^WT^* GBM cases relative to PyroSeq methylation percentage score. Data are presented as the percent of cases prevalent in MGMTai and PyroSeq scoring buckets representing concordance between the AI model and PyroSeq assay. Data are normalized by PyroSequencing bucket. MGMT, O^6^-methylguanine-DNA methyltransferase; PyroSeq, pyrosequencing.

Following the algorithm lock of MGMTai in February 2025, we conducted a prospective validation study to assess the model’s performance in real-world clinical settings. Over the course of 9 months (until November 2025), we collected prediction results from 3464 cases with PyroSeq results. A total of 2772 are *IDH*^WT^ and, of those, 1870 are predicted in the lowest or highest scoring buckets (model score ≤0.2 or >0.8). The model demonstrated robust performance with a PPV of 86.4% and a NPV of 95.3%. To further characterize the relationship between model predictions and PyroSeq, we generated a prevalence heatmap (Figure 4) that visualizes the distribution of outcomes across the MGMTai model score spectrum and PyroSeq percentage with normalization by row. Two distributions are noted (yellow and red shading) where the MGMTai model exhibited the ability to predict MGMT^met^ status. In concordance with PyroSeq methylation percentage of <7%, representing “Not Methylated” *MGMT* (Figure 4), for all samples with a PyroSeq score of 1%, 79.6% of cases were assigned to the [0-0.2) MGMTai bucket. Similarly, samples with PyroSeq scores of 2%-3% had 64.7% assigned to the [0-0.2) MGMTai bucket. Also present was the upper limit of methylation detection at PyroSeq methylation percentages (≥9%) representative of hypermethylation, with scores reaching a maximum of 75.5% of cases also assigned to the corresponding MGMTai bucket of [0.8-1). All other bucketing prevalence comparisons fell below 10.6%, providing additional evidence for the model’s discriminative capacity and supporting its integration into clinical decision-making workflows. Validation matrix for molecularly defined GBM is presented in Figure S5.

## Discussion

The application of machine learning and AI to oncology questions has produced dramatic advances in diagnosis and patient management.^19–22^ In the context of molecular characterizations, machine learning has enabled the field to comprehensively characterize hallmark cancer-associated genes, guiding treatment decisions on a patient-by-patient basis. Glioblastoma patients require advancements of this scope to identify novel predictive and prognostic indicators that lead to urgently needed improvements in treatment efficacy and survival. Our work explores a possible improvement in efficiency and reliability of *MGMT* methylation status determination by implementing machine learning techniques and, to the best of our knowledge, is the first use of AI in *MGMT*^met^ classification in a large clinical cohort with available sequencing data.

MGMTai utilizes the large clinico-genomic database at Caris Life Sciences to robustly classify patient *MGMT*^met^ status and better predict survival outcomes in TMZ-treated patients compared to traditional methods. *MGMT^met^* is a reliable predictor of GBM response to TMZ therapy, where concurrent TMZ and radiotherapy has been the standard of care for nearly 2 decades.^23^^,^^24^ Traditional methods of expression and methylation levels include MSP and PyroSeq, each of which is beset by unique limitations that may convolute data interpretation or require relatively large tumor specimens for proper evaluation.

MSP, either qualitative MSP or qMSP, is an original resource for methylation detection, first described in 1996.^25^ Although MSP continues to offer some clinical benefit, advances in methylation detection methods allow improved consistency, and machine learning-assisted classification algorithms provide enhanced precision and reliability in predicting clinical outcomes, as we have shown using MGMTai. The qualitative nature of MSP and the heterogeneity of MGMT methylation in tumor tissue contribute to its inconsistency.^8^^,^^26^ Further detracting from the utility of MSP is the large quantity of tumor tissue required for analysis.^26^^,^^27^ qMSP mitigates some of the qualitative drawbacks of MSP, but this potential benefit is reduced by the existence of an “equivocal” category and by the uncertainty of methylation cutoff values overall.^8^ Similar issues with *MGMT*^met^ heterogeneity also pertain to qMSP, reducing testing sensitivity and generating a patient subpopulation that may not be appropriately treated.

Pyrosequencing is another quantitative assay that can be considered a historic standard, but like qMSP, is plagued by numerous limitations that may be resolved through the implementation of machine learning within genetic databases. As with MSP, assigning methylation status cutoff values with PyroSeq remains fraught. Numerous reports indicate differing parameters for determining methylation status cutoff values, with common values of 8%^28^ and 10%^29^ as well as literature-reported values of 5%, 9%, and 11%.^29–34^ Our in-house PyroSeq method values draws the “methylated” cutoff at 8%, with values above 6% considered equivocal as the best fit values in our cohort. These inconsistencies in designating precise methylation cut points have potentially critical implications for predicting patient outcomes and designing treatment plans. There are also substantial financial consequences to utilizing PyroSeq, which is considerably more expensive than traditional MSP.^35^ In addition, the minimum tumor content required for PyroSeq is 50%, whereas only 20% tumor content is required for the NGS assay used by MGMTai. This factor alone may eliminate a number of patients who could benefit from PyroSeq but whose available specimen does not have adequate tumor content.

Our methylation assessment model, MGMTai, created by comparing a vast amount of currently available patient genetic data linked to insurance claims information, accurately scores methylation status in GBM patients. In our LightGBM model, we captured and described the top 100 molecular features contributing to MGMTai classification performance (Table S1), though the importance of *MGMT* expression levels cannot be understated, as shown by the expression-dependent survival trends in Figure 2B and highlighted by previous literature.^36^ While it should be acknowledged that leading features including *SUFU* expression,^37^  *PLCG2* expression,^38^ and *SNX29* expression,^39^ among others, play vital oncogenic or suppressive roles in numerous cancers, *MGMT* expression alone carried the largest weight in developing the MGMTai model. MGMTai shows a high level of methylation predictability while also permitting distinct and sensitive methylation status cutoff values tightly linked to patient OS. Related to these distinct cutoff values, our model reports 27.5% of our patient cohort yielding scores that would be categorized as “hypermethylated” *MGMT*^met^ status via PyroSeq. Though literature reports approximately 40% of *IDH1/2^WT^* GBM patients will be *MGMT*^met^, we can attribute this discrepancy to the lack of a definitive methylation classification cutoff found in PyroSeq and our selected exclusion criteria for patient samples, possibly removing samples that may contribute to balancing our values with what is shown in literature.^40^

We also show that MGMTai can reliably predict TMZ-treated GBM patient survival. Our model mirrored mOS results shown for PyroSeq when comparing MGMTai buckets, where scores >0.8, representing “hypermethylated” according to PyroSeq, yielded significantly longer mOS in patients treated with TMZ. In fact, all comparisons with increasing MGMTai scoring yielded longer mOS compared to lower MGMTai scores. This result supports the reliability of MGMTai to replicate traditional PyroSeq patient outcomes data, while capturing *MGMT*^met^ in a more granular classification system. Further, we showed that the MGMTai model can prospectively cast *MGMT*^met^ status classifications, with strong PPV and NPV performance. Nearly all data captured and reported by MGMTai were completed in less than 10 days, in line with NGS assay turnaround time for Caris Life Sciences in 2025.

There are, however, some important limitations to our study. First, some prognostically relevant patient information (eg extent tumor resection) was not available to us for inclusion in our multivariate model. Second, some tumor specimens from potentially study-eligible patients were not included because of unavailable survival data. While we have no reason to suspect that lack of clinical information is associated with patient survival, we cannot exclude this possibility with absolute certainty. Third, there are additional important molecular alterations at play within the GBM environment outside of *MGMT* that may influence TMZ response and OS. Finally, we have restricted our study population to *IDH^WT^* GBM in the training and testing of the MGMTai model. We have done this in order to construct a patient cohort that is as close to a “pure” GBM population as possible. In pursuit of this goal, we have also excluded (1) GBMs in patients younger than 50, (2) all *IDH*-mutant gliomas, and (3) *IDH^WT^* gliomas that the newest WHO classification system would not designate as GBM (for instance, H3-altered pediatric high-grade gliomas).^41^ These patient populations should also be analyzed to develop a fully robust model.^42^^,^^43^ Despite these potential limitations, our model overcomes important drawbacks of traditional *MGMT* methylation diagnostic methods (including reliability, expense, and challenging specimen requirements) while producing improved survival predictions. Application of our approach to the evaluation of other tumor histologies (eg low-grade glioma), TMZ efficacy prediction in other cancer models, and further genetic characterizations outside of *MGMT*, including *EGFR* amplification and *pTERT* mutation, are underway.

## Conclusion

This work represents a proof-of-concept, describing a novel machine learning algorithm developed that efficiently classifies *MGMT* methylation status compared to the traditional PyroSeq method. We demonstrate that MGMTai consistently stratifies *IDH*^WT^ GBM patients by *MGMT* methylation status in a manner concordant with PyroSeq classification buckets with impressive PPV and NPV. In fact, MGMTai draws more distinct classification cutoff values and provides clearer separation of patient survival curves than *MGMT*^met^ classification, outperforming PyroSeq and other *MGMT*^met^ classification assays. Although this work demonstrates the potential of AI involvement across molecular classification, replacement of traditional methods is not yet warranted. Further validation in larger, independent cohorts will be essential to strengthen these claims made in this work and to further improve *MGMT*^met^ classification algorithms.

## Supplementary Material

vdag103_Supplementary_Data

## Data Availability

The datasets generated during and/or analyzed during the current study are available from the corresponding author on reasonable request. The deidentified sequencing data are owned by Caris Life Sciences. Qualified researchers can apply for access to these summarized data by contacting the corresponding author (tnicolaides@carisls.com) and signing a data usage agreement.

## Code Availability

The code generated during the current study are available from the corresponding author on reasonable request. Code will be made available for replication and verification purposes from the corresponding author upon signing a code usage agreement and subject to company approval.
