# Supplementary material for: MGMTai: O6-methylguanine-DNA methyltransferase (MGMT) methylation prediction in isocitrate dehydrogenase (IDH)-wild type glioblastoma to direct temozolomide therapy
Source: Neurooncol Adv. 2026 Apr 16;8(1):vdag103. doi: 10.1093/noajnl/vdag103 (PMC13161554; doi:10.1093/noajnl/vdag103)
Supplement: vdag103_Supplementary_Data [file vdag103_supplementary_data.docx]

**Supplementary Figure 1 – Expression normalization and transformation of molecular data.** CAP/CLIA validated percentile-based RNA expression normalization to combine expression data from WTS and MI Tumor Seek Hybrid™ **(A)**. Transformation of WTS RNA expression to MI Tumor Seek Hybrid™-like values for confirmation of input data type in clinical setting **(B)**. AUC – Area under the curve.


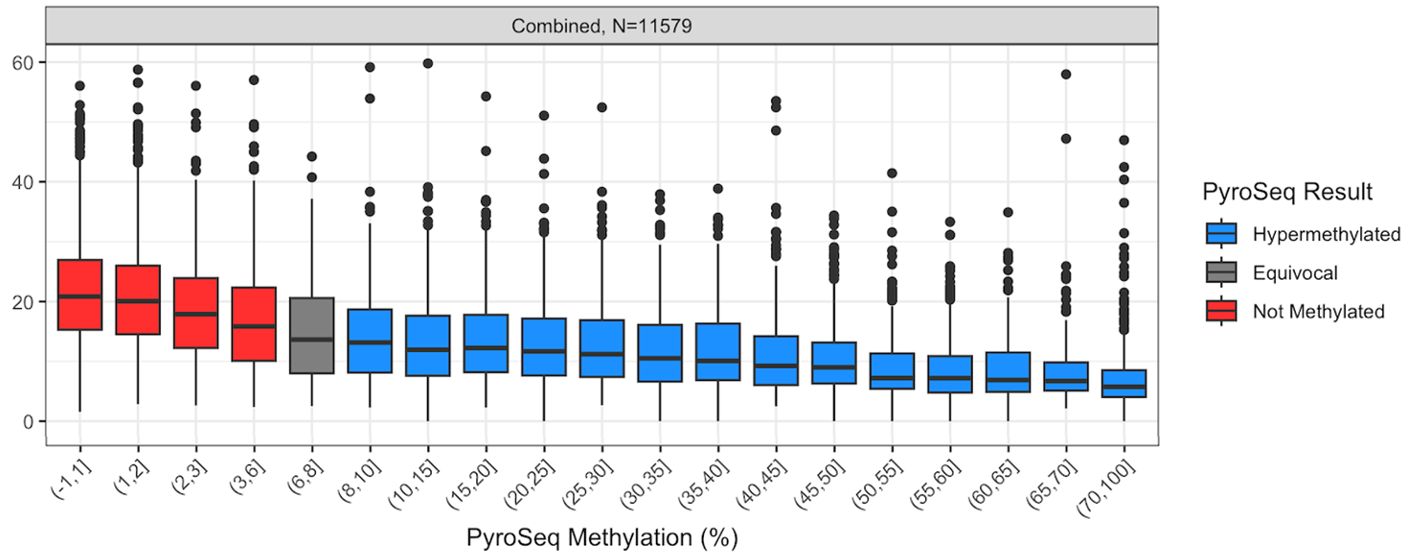


**Supplementary Figure 2 – *MGMT^met^* percentage across combined GBM cohort analyzed by PyroSeq relative to *MGMT* RNA expression.** Methylation status cutoff values at <6% (not methylated, red), 6-8% (equivocal, gray), and >8% (hypermethylated, blue).


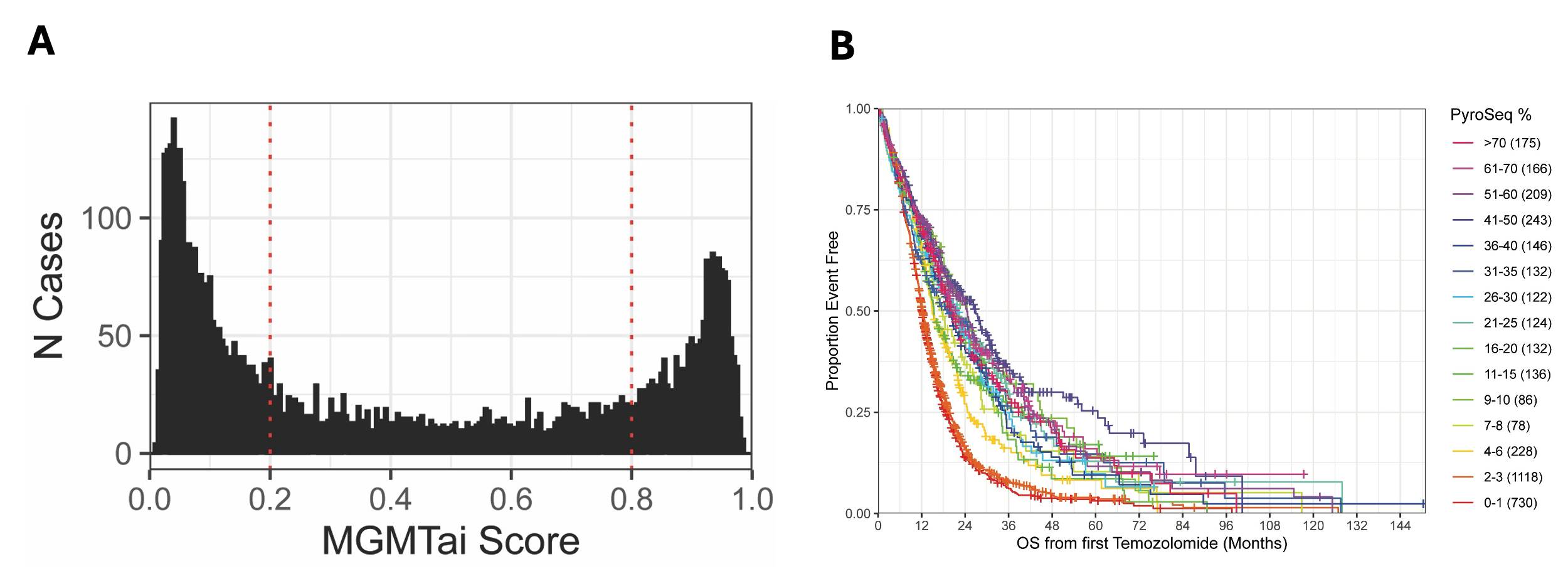


**Supplementary Figure 3 – Distribution of GBM cases by MGMTai score and OS from first TMZ by PyroSeq Methylation Percentage.** GBM cases were distributed across MGMTai scores in a classical U-shaped distribution with statistically different TMZ responses (A). GBM cases were also examined for OS from first TMZ by PyroSeq MGMTmet percent by decile (B).


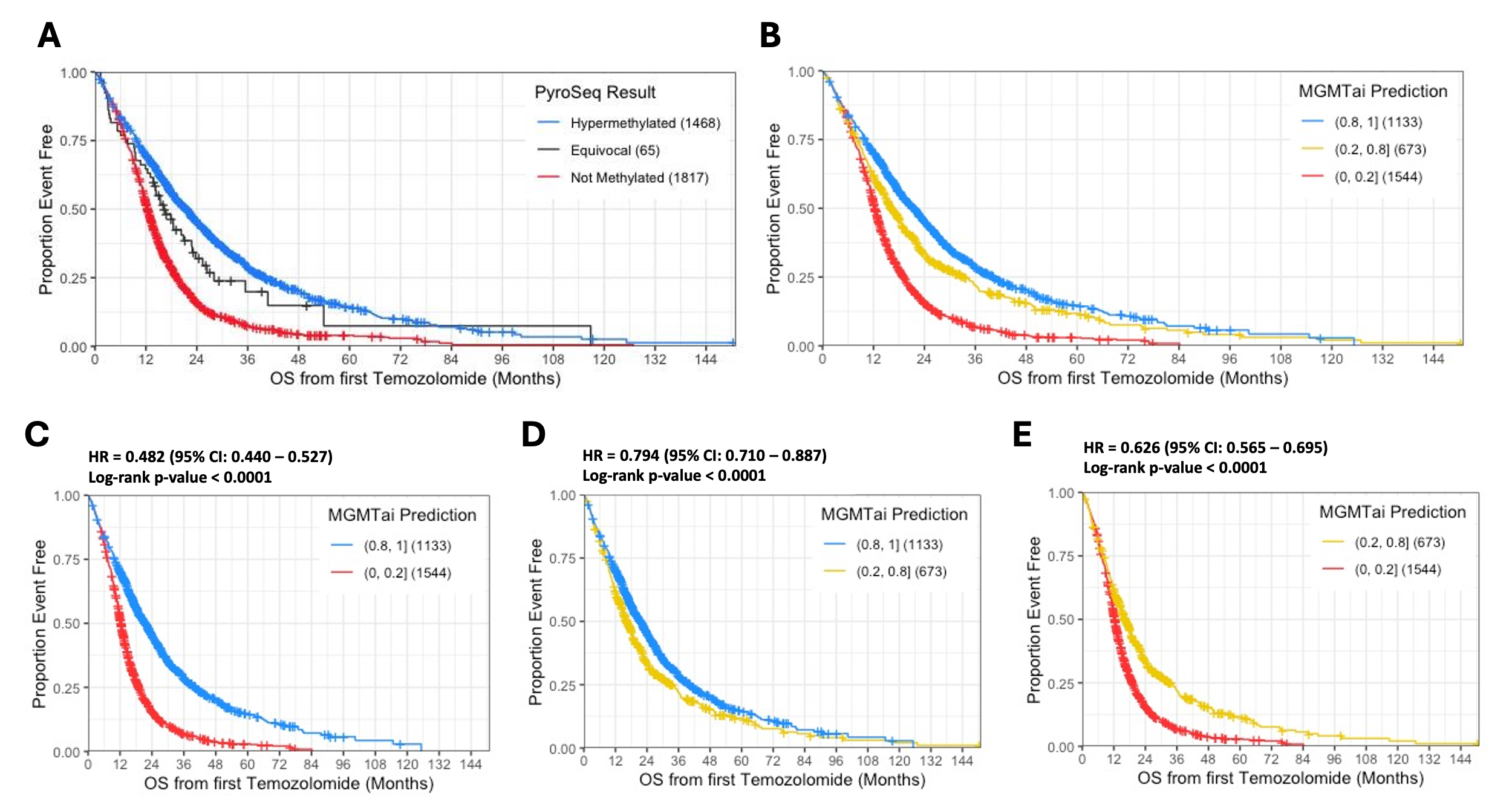


**Supplementary Figure 4 – Overall survival (OS) from first temozolomide (TMZ) treatment by traditional pyrosequencing or MGMTai in “pure” GBM.**“Pure” GBM determined by presence of *TERT* mutation, *EGFR* amplification, and/or Chr +7/-10 modification. OS in months post-TMZ by *MGMT^met^* status determined by pyrosequencing as hypermethylated, equivocal, or not methylated **(A)** or MGMTai as methylation score buckets **(B)**. OS comparison between MGMTai predicted methylation score buckets (0.8, 1] and (0, 0.2] **(C)**, (0.8, 1] and (0.2, 8] **(D)**, (0, 0.8] and (0, 0.2] **(E)**. Statistical significance where p < 0.05.


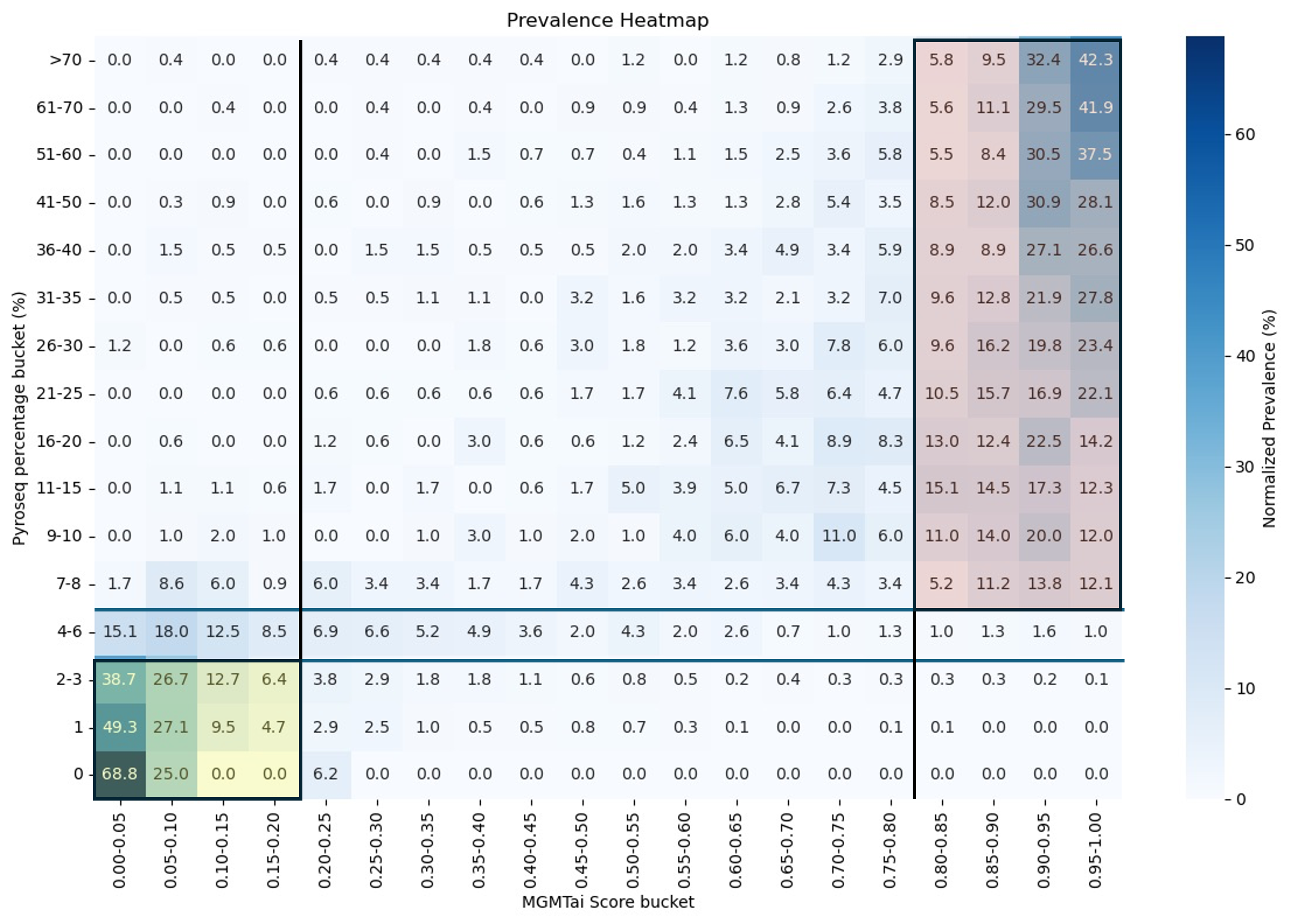


**Supplementary Figure 5 – Prospective validation of MGMTai by case prevalence relative to PyroSeq in “pure” GBM.**

“Pure” GBM determined by presence of *TERT* mutation, *EGFR* amplification, and/or Chr +7/-10 modification. MGMTai prospectively predicted methylation score of 2,772 *IDH^WT^* GBM cases relative to PyroSeq methylation percentage score. Data are presented as the percent of cases prevalent in MGMTai and PyroSeq scoring buckets representing concordance between the AI model and PyroSeq assay. Data are normalized by PyroSequencing bucket.

| **Supplementary Table 1 – Top 100 molecular features in MGMTai model development.** | | | |
| --- | --- | --- | --- |
| **Gene** | **Feature Type** | **Gene** | **Feature Type** |
| *MGMT* | Expression | *MLLT10* | Expression |
| *SUFU* | Expression | *APLNR* | Expression |
| *PLCG2* | Expression | *BMPR1A* | Expression |
| *NCOA4* | Expression | *NF2* | Copy Number |
| *WAS* | Expression | *FANCM* | Copy Number |
| *SNX29* | Expression | *MAF* | Expression |
| *TAL1* | Expression | *DNMT3A* | Expression |
| *ERCC6* | Expression | *HRAS* | Expression |
| *STAT6* | Expression | *MYCN* | Expression |
| *IKBKE* | Expression | *ACKR3* | Expression |
| *GRM3* | Expression | *FANCG* | Expression |
| *BTG2* | Expression | *ERBB3* | Expression |
| *ZBTB16* | Expression | *CACNA1D* | Expression |
| *CSF3R* | Expression | *GREM1* | Expression |
| *NFKB2* | Expression | *CEBPA* | Expression |
| *IKZF1* | Expression | *LMO2* | Expression |
| *FGFR2* | Expression | *TFEB* | Expression |
| *PRKCH* | Expression | *ARID1A* | Copy Number |
| *LMNA* | Expression | *CYP17A1* | Expression |
| *ASPSCR1* | Expression | *TSC2* | Expression |
| *INPP4B* | Expression | *BCL2* | Expression |
| *MYH11* | Expression | *CUX1* | Expression |
| *SOX10* | Expression | *SH2B3* | Expression |
| *VTI1A* | Expression | *MAP2K2* | Expression |
| *CDKN2A* | Expression | *PMS2* | Copy Number |
| *TYRO3* | Expression | *TNFAIP3* | Expression |
| *CDKN2B* | Expression | *NT5C2* | Expression |
| *FOXP1* | Expression | *PTPRT* | Expression |
| *BCL2L2* | Expression | *CDH23* | Expression |
| *HDAC1* | Expression | *ZNF521* | Expression |
| *PTEN* | Expression | *SLIT2* | Expression |
| *AIP* | Expression | *ERRFI1* | Expression |
| *GNA13* | Expression | *GPC3* | Expression |
| *CD22* | Expression | *RPN1* | Expression |
| *POLD4* | Expression | *AKT1* | Expression |
| *TGFBR2* | Expression | *NOTCH1* | Expression |
| *AXL* | Expression | *PLAG1* | Expression |
| *NKX2-1* | Expression | *EZR* | Expression |
| *TSHZ3* | Expression | *CTNNA1* | Expression |
| *MUS81* | Expression | *EBF1* | Expression |
| *INHBA* | Expression | *ABCB11* | Expression |
| *TEK* | Expression | *IKZF1* | Copy Number |
| *GATA1* | Expression | *TRAF3* | Expression |
| *SMO* | Copy Number | *PDGFB* | Expression |
| *RPA4* | Expression | *NYNRIN* | Expression |
| *FOXO4* | Expression | *P2RY8* | Expression |
| *MAPK3* | Expression | *EZH2* | Copy Number |
| *KDM6A* | Expression | *SMARCA4* | Expression |
| *LDLR* | Expression | *BCL2L1* | Expression |
| *ITK* | Expression | *RHOA* | Expression |
